# The Usefulness of Percutaneous Endoscopic Technique in Multifocal Lumbar Pathology

**DOI:** 10.1155/2019/9528102

**Published:** 2019-01-03

**Authors:** Chul-Woo Lee, Kang-Jun Yoon

**Affiliations:** Department of Neurosurgery, St. Peter's Hospital, Seoul 135-809, Republic of Korea

## Abstract

*Introduction. *The multifocal lumbar pathology including disc herniation and stenosis in the spinal canal or foramen has been considered the most difficult to approach surgically. It often requires mandatory dual approaches and/or fusion techniques. Traditional percutaneous endoscopic lumbar transforaminal and interlaminar approach has been focused on unifocal disc herniation. However, the development of endoscopic spinal instruments and surgical technique has broadened surgical indication and therapeutic boundary in endoscopic spine surgery.* Cases Presentation. *The authors present outcomes of four patients with multilumbar pathology including highly inferior migrated disc combined with lateral recess stenosis, multifocal disc herniation, bilateral disc herniations in spinal canal and foraminal disc herniation combined with central canal stenosis. They were successfully treated by percutaneous uniportal full endoscopic approach with single incision.* Conclusion.* Percutaneous endoscopic spine surgery is a safe and effective tool to figure out multilumbar pathology in a minimal invasive way.

## 1. Introduction

Traditional percutaneous endoscopic lumbar transforaminal and interlaminar approach has been focused on unifocal disc herniation [1–5]. However, the evolution of endoscopic instruments such as drills and punches and the development of surgical technique have broadened surgical indication and therapeutic boundary in endoscopic spine surgery. Lumbar spinal diseases ranging from simple contained disc to complicated cases such as highly migrated disc herniation and other pathology combined with bony degeneration to produce foraminal and canal stenosis can now be operated fully with endoscope using various accesses and techniques [6–13].

The multifocal lumbar pathology including disc herniation and stenosis in the spinal canal or foramen has been considered the most difficult to approach surgically. It often requires mandatory dual approaches and/or fusion techniques. Endoscopic surgical techniques may reduce the need for these more invasive methods. A uniportal full endoscopic approach with single incision can satisfactorily resolve these challenging cases. Here we present outcomes of four patients with multipathologies in the lumbar spine who were successfully managed with a single endoscopic approach.

## 2. Cases Presentation

### 2.1. Case 1

**Figure 1:**
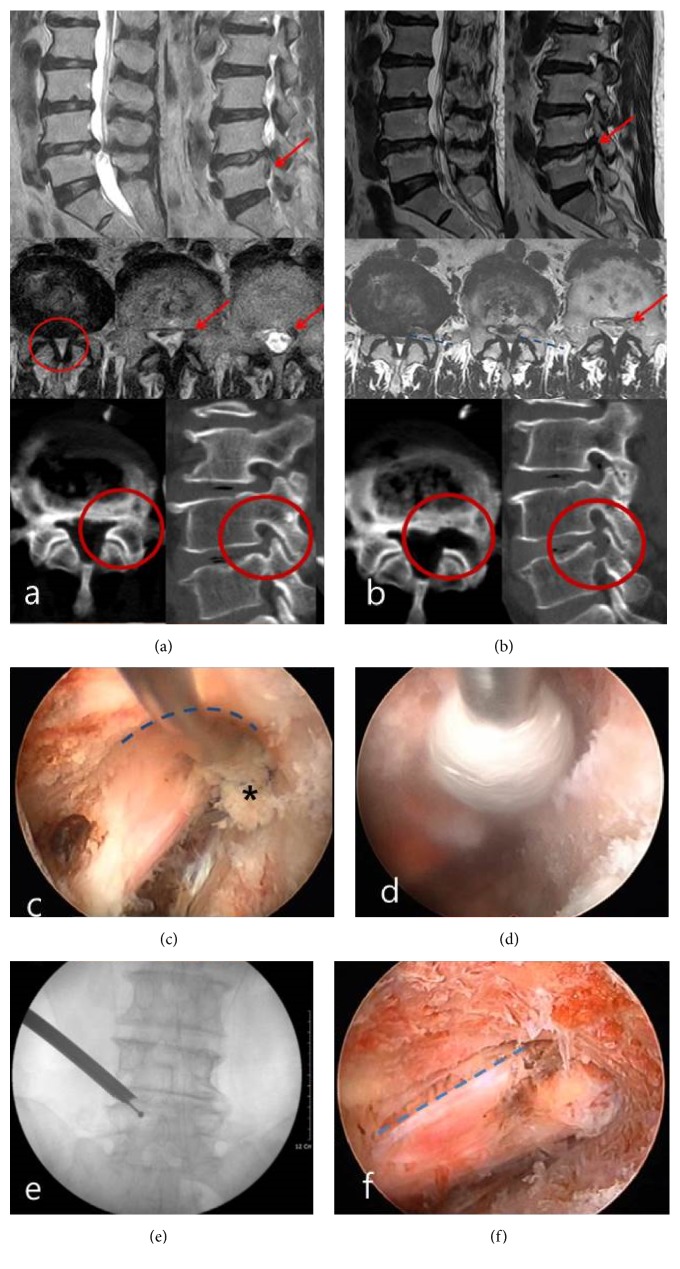
Highly inferior migrated disc combined with spinal canal and lateral recess stenosis. (a) Preoperative sagittal and axial T2-weight MRI showing ruptured right side inferior migrated disc material combined with central canal and lateral recess stenosis at L4-5. Red arrow: inferior migrated disc; red circle: stenotic region in spinal canal and lateral recess. (b) Postoperative axial and sagittal T2-weight MRI and CT showing the removed inferior migrated disc materials and decompression of lateral recess at L4-5. Red arrow: decompressed area by removal of inferior migrated disc materials; red circle: decompressed area from preoperative stenosis; blue dotted line: resected plane of superior articular process. (c) Part of inferior migrated disc materials was seen by retraction of flexible probe. Dotted blue line: resected ventral plane of superior articular process by reamers; asterisk: tip of inferior migrated disc materials. (d) Further decompression of lateral recess was performed by drilling. (e) Intraoperative C-Arm image showing the location of drill tip during lateral recess decompression. (f) Totally decompressed traversing root was seen at the end stage of operation blue dotted line: dorsal margin of the traversing root.

A 60-year-old woman suffered from left gluteal, thigh, and calf pain along the L5 dermatome for two months. Manual muscle test for the left great-toe dorsiflexion and the ankle dorsiflexion showed grades III and IV, respectively. She also suffered from neurogenic intermittent claudication symptom (50 m). Magnetic resonance (MR) images demonstrated disc extrusion and downmigrated disc herniation combined with spinal canal and lateral recess stenosis at L4–5 level (Figure 1(a)). Although she underwent a steroid epidural injection with medications, the pain did not improve. Foraminoplastic percutaneous endoscopic lumbar discectomy (PELD) using reamers was performed in the prone position under local anesthesia [6]. The patient communicated with the surgeon during the entire procedure. The blue stained inferior migrated ruptured disc was seen beyond the partially resected superior articular process (SAP) (Figure 1(e)). The herniated disc and fibrotic scar tissues were released and removed using endoscopic forceps and radiofrequency. The ventral portion of decompressed traversing root was confirmed. Additional removal of SAP was performed. Part of the L5 upper end plate around the lateral recess was drilled out. The ligament flavum was also removed, reaching the spinal canal by an endoscopic punch (Figures 1(c) and 1(d)). This resulted in the whole traversing root being exposed (Figure 1(f)). After the operation, her visual analogue scale (VAS) scores of the back and leg pain improved from 6 and 8, respectively, to 2 and 1, respectively. Postoperative MR and CT images (Figure 1(b)) showed complete removal of the ruptured disc fragment and decompressed lateral recess area. The patient was discharged on the day after PELD.

### 2.2. Case 2

**Figure 2:**
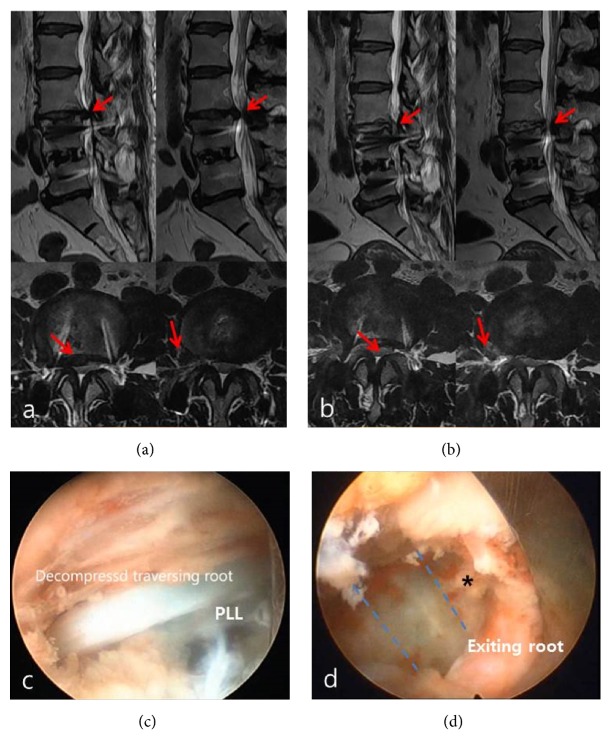
Single level multi-focal, paracentral, and far-lateral lumbar disc herniations. (a) Preoperative sagittal and axial T2-weight MRI showing multifocal, paracentral, and far-lateral lumbar disc herniations at L3-4. Red arrows: paracentral and extraforaminal disc herniation. (b) Postoperative axial and sagittal T2-weight MRI showing the removed paracentral and far-lateral lumbar disc herniations at L3-4. Red arrows: decompressed area by removal of paracentral and extraforaminal disc herniation. (c) Paracentrally herniated disc materials were removed and decompressed traversing root was seen. PLL: posterior longitudinal ligament. (d) Change of working cannula angle showing the foraminal area. Asterisk: tip of remnant extraforaminal disc; dotted blue line: disc level.

A 50-year-old woman visited the clinic because of severe right-leg radiating pain along the L2 and L3 dermatome. She has a history of fusion surgery five years ago. MR images revealed intracanal and extraforaminal multifocal soft disc herniation at the L3-4 level (Figure 2(a)). Although she underwent nerve-root block at L3 and L4, the pain sustained. PELD with foraminoplasty using reamers was performed. After removal of the herniated disc in the paracentral area (Figure 2(c)), working cannula was slightly withdrawn and reapproached with a stiff angle in order to confirm compressed exiting root. Another stained ruptured disc fragment was found at the axilla area of exiting root by a gentle circular twisting motion of working cannula (Figure 2(d)). It was removed by forceps with caution to avoid the exiting root injury by excessive manipulation. Postoperatively, the patient's preoperative leg pain was resolved without complications. Back and leg pain VAS scores decreased from 6 and 7 preoperatively to 3 and 2 postoperatively. MR images showed successful simultaneous removal of paracentral and extraforaminal double disc herniations (Figure 2(b)).

### 2.3. Case 3

**Figure 3:**
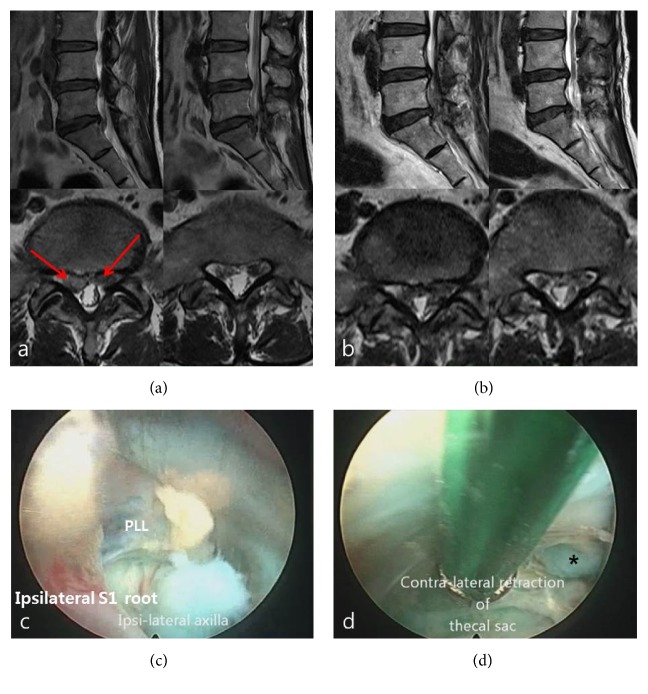
Bilateral disc herniations on L5-S1. (a) Preoperative sagittal and axial T2-weight MRI showing thecal sac compression due to L5–S1 bilateral disc herniations. Red arrows: bilateral disc herniations compressing thecal sac. (b) Postoperative axial and sagittal T2-weight MRI showing decompressed thecal sac and bilateral S1 root by removal of bilateral disc herniation at L5-S1. (c) Ruptured disc material seen at ipsilateral axilla area of S1. PLL: posterior longitudinal ligament. (d) Tip of contralateral ruptured disc was exposed by retraction of thecal sac. Asterisk: tip of the ruptured disc from contralateral epidural space.

A 58-year-old woman presented with acute onset left-leg radiating pain. She also had constant right-leg radiating leg pain for one year. Bilateral straight leg raise test was positive. MR images showed L5–S1 bilateral herniated disc (Figure 3(a)). Despite conservative treatment with physical therapy and interventional pain management, the patient's symptom did not improve. A working cannula was placed on the interlaminar space via a 0.7 mm skin incision under epidural anesthesia. The ligamentum flavum was then split by the probe in the middle part on the ipsilateral side. A working cannula with endoscope was subsequently introduced into the epidural space through the split ligamentum flavum and the dura sac and nerve root were exposed. After gentle retraction of the ipsilateral S1 root, epidural dissection by various endoscopic instruments, a working channel was inserted into the axillary area of S1 root. Sequestrated disk materials located on the ipsilateral side were found and removed with forceps (Figure 3(c)). The central portion of the annulus and the posterior longitudinal ligament (PLL) located at the center were cleared and identified to expose the contralateral side. Further exposure of contralateral epidural space by retraction of thecal sac was followed. Another protruded disc was identified under thecal sac on the contralateral side (Figure 3(d)). Probes were moved to the site to remove and puncture organized disc materials. Forceps were used to remove the contralateral ruptured disc. The working cannula was withdrawn and reapproached over the thecal sac to observe the contralateral side. Decompressed contralateral the traversing nerve root was confirmed. Postoperatively, the patient showed no symptoms radiating to the legs. There were no deficits on neurological examination. Postoperative MR images revealed that preoperative herniated discs were successfully removed bilaterally (Figure 3(b)).

### 2.4. Case 4

**Figure 4:**
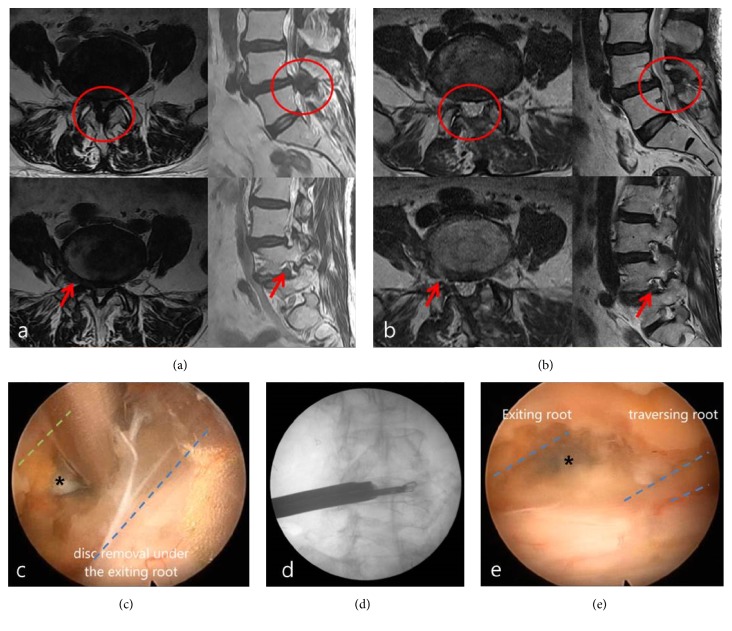
Extraforaminal disc herniation combined with central canal stenosis. (a) Preoperative sagittal and axial T2-weight MRI showing foraminal disc herniation combined with central canal stenosis. Red arrows: extraforaminal disc; red circle: stenotic region in spinal canal. (b) Postoperative axial and sagittal T2-weight MRI showing decompressed thecal sac and contralateral L4 root after removal of extraforaminal disc herniation at L4-5. Red arrows: decompressed area by removal of extraforaminal disc; red circle: enlarged spinal canal from preoperative stenosis. (c) Contralateral extraforaminal disc seen under the contralateral exiting root. Asterisk: ruptured extraforaminal disc; green dotted line: inferior margin of contralateral exiting root; blue dotted line: contralateral margin of thecal sac. (d) Intraoperative C-Arm image showing the location of forceps grasping protruded contralateral extraforaminal disc materials. (e) Decompressed both contralateral exiting and traversing root. Asterisk: removed site of extraforaminal disc. Blue dotted line: contralateral exiting and traversing root.

A 77-year-old woman presented with complaints of radicular pain in the right gluteal region and anterolateral aspect of her thigh and leg for three months. She was also suffering from neurogenic claudication symptom. She could not walk more than 50 meters continuously. MR images of the lumbar spine revealed extraforaminal disc combined with central canal stenosis on L4-5 (Figure 4(a)). A plain radiograph showed a minimal listhesis. The L4-5 segment was stable. The patient was operated under epidural anesthesia in a prone position on a spinal frame. The skin incision was marked lateral to spinous process contralateral to the side of the foramen to be decompressed and directed towards the side of the stenosis. A 12 mm working cannula was placed on the lower margin of L4 ipsilateral spinolamina junction initially and an endoscope was inserted. Laminotomy was performed with high-speed endoscopic drills. Thinned-out lamina was adequately removed with an endoscopic Kerrison rongeur. The base of the spinal process was then removed to obtain a clear view of the contralateral lateral recess and the foramen. The ligamentum flavum was initially preserved to protect the dura. After completion of bony resections, the ligamentum flavum was removed piecemeal starting from the midline. Lateral margin of thecal sac was exposed. Gentle retraction of the contralateral thecal sac from the lateral to medial direction revealed a protruded contralateral side extraforaminal disc which was removed by endoscopic forceps (Figures 4(c) and 4(d)). Afterward, the opposite lateral recess and the foramen were further decompressed by removing the ligamentum flavum, drilling osteophytes, clearing all disc fragments, and undercutting the medial facet. Finally, successfully decompressed contralateral exiting and traversing nerve root was confirmed (Figure 4(e)). After the operation, her VAS scores of the back and leg pain improved from 5 and 8 preoperatively to 2 and 2, respectively. Postoperative MR images showed complete removal of ruptured extraforaminal disc fragments and decompressed spinal canal (Figure 4(b)).

## 3. Discussion

Spinal disease is the natural aging process. Such degenerative change induces lumbar spinal disease which has a variable spectrum ranging from simple disc herniation to severe degenerative spondylosis such as listhesis, stenosis, and kyphoscoliotic deformity. Among those diseases, a simple single lumbar pathology could be mostly figured out by a single therapeutic modality and approach. However, multifocal or combined different type of pathology in the lumbar spine would need more invasive surgical methods such as two staged dual approaches or fusion technique in order to solve such different and complex combined pathologies.

We presented outcomes of four patients with multilumbar pathology who were successfully treated by a single endoscopic approach. If endoscopy was not used, more invasive treatments would have been needed for these cases.

Endoscopic operations such as arthroscopy and laparoscopy are becoming standard operations nowadays. Lumbar spinal diseases ranging from simple contained disc to complicated cases such as highly migrated disc herniation and other pathology combined with bony degeneration to produce foraminal and canal stenosis can now be treated with full-endoscopic surgery using various accesses and techniques [6–13]. Many authors have reported advantages of percutaneous endoscopic surgery compared to previous traditional surgery. These advantages include minimal injury to spinal segmental structures including muscle, facet joint and dorsal ramus, short hospital stay, early return to regular activity, and patient's high satisfaction [7, 14, 15]. Numerous merits of percutaneous endoscopic surgery were also revealed distinctly in the current series. Traditional spinal surgery needs massive paravertebral muscle dissection and two staged operations in order to acquire enough operative fields to cover different and distant dual pathologies like current cases. However, percutaneous endoscopic approach achieved the same goal with only 7-12 mm single tiny skin portal and minimized handling of endoscopic instruments. All patients were discharged within one or two days after the operation. Postoperatively, patients immediately resumed their regular activities of daily living. They were able to return to clerical forms of work within seven days. Such postoperative course might not be observed if we operated with traditional surgical methods to treat these cases. Successful clinical results in these multilumbar pathology cases mentioned above might be due to some unique characteristics of endoscopic spine surgery.

The lens located on the tip of the tube-shaped endoscope to see the operative field can be referred to as the “operative eye”. The “operative eye” can be placed very close to the operative target directly passing anatomical structures in endoscopic spine surgery. Unlike traditional bare-eye or microscopic surgery, it is not necessary to destroy much of normal structures to access the target pathology and secure operative corridor to see the operative field. In the first case, traditional laminotomy techniques could be used for lateral recess decompression with removal of the highly inferior migrated disc herniation. However, these surgical options could not avoid injury to posterior segmental structures by dissection of the paravertebral muscle and the partial removal of the lamina and facet joint. Target oriented direct accessibility in endoscopic surgery mentioned above helped us minimize the operative iatrogenic injury and save normal segmental spinal structures in current cases. It led to good clinical outcome such as patient's fast recovery and early return to regular activity despite their multilumbar pathology.

Operative instruments used in endoscopic spine surgery are relatively small compared to near anatomical spinal structures. The “operative eye” on the tip of such a small endoscope can navigate around the target with minimal pivoting movement of the endoscope via an initial single skin portal. Moreover, recent development of the endoscopic drill system has expanded surgical boundary where the endoscope could not previously approach or move around. Such characteristics of endoscopic spinal surgery provided probing and small sized working spot, enabling authors to explore a relatively large operative field and manage two different distant targets simultaneously in current cases. The second and third cases showed navigability of the spinal endoscope that helped us treat the multilumbar pathology successfully.

Variability of endoscopic surgical angle is another distinct feature of spinal endoscope regarding surgical success in current series. Only a slight withdrawal of endoscope after the initial approach can give surgeons the opportunity to reapproach, change the working trajectory, and manipulate structures around different surgical targets without needing another skin incision or different secondary surgical corridor as shown in the second and third cases. The optical angle of a spinal endoscope is 15-20 degrees. With rotation or tilting of the endoscope, an endoscopic operative angle can provide a more variable surgical view and working trajectory. It helps the surgeon reach farther targets that could not be reached by microscope. It also helps surgeons explore hidden areas easily without destroying normal anatomical structures needed to be removed to observe the target with traditional surgical methods. The fourth case was a good example of endoscopic surgery which was performed by a precise, targeted approach via the least invasive surgical route using the endoscopic angled and long-distance visibility. Instead of using dual approach for both spinal canal decompression and removal of extraforaminal disc herniation or the fusion method with wide decompression, endoscopic contralateral approach was chosen. It achieved the same surgical goal. Optimized oblique sublaminoplasty for canal decompression and removal of far lateral disc from contralateral side were possible due to the long distance of the visibility, natural optical angle, and tilting maneuver of the spinal endoscope.

In the current case series, satisfactory clinical results were acquired from all patients by using minimally invasive endoscopic procedure. However, percutaneous endoscopic spine surgery is not omnipotent. Although all cases in the current series were successfully resolved by a single endoscopic approach, these techniques cannot be applied to all forms of lumbar spinal diseases, especially for those with severe lumbar spinal stenosis with spondylolisthesis or instability. It should be performed carefully for selected patients. Percutaneous endoscopic spine surgery has a steep learning curve as previously reported by many authors [7, 16–19]. All cases in the current series were operated by a single surgeon (C.W. Lee) who has performed over 2,000 cases of endoscopic spine surgeries. Lack of clinical experience in endoscopic spine surgery could be the major cause of surgical failure and undesirable operative complications [17, 20–22]. Furthermore, complex and difficult cases with multilumbar pathology would have much higher operative failure risk. Endoscopic surgeons who are considering the use of endoscopic technique in treating multilumbar pathology are recommended to have abundant experience in endoscopic surgery. They also should be familiar with the usage of various endoscopic instruments (such as endoscopic drills and reamers) and working cannula handling (such as withdrawal, tilting, and rotation).

## 4. Conclusions

Percutaneous endoscopic spine surgery is a safe and effective tool to resolve multilumbar pathology in a minimal invasive way. It can be an alternative to traditional surgical methods by minimizing iatrogenic injury to normal segmental structures and providing good clinical outcome to the patients.

## Data Availability

The data used to support the findings of this study are available from the corresponding author upon request.
